# Controlled Catalysis Delivering High Molecular Weight Polyesters as Recyclable Alternatives to Polystyrenes

**DOI:** 10.1002/anie.202505070

**Published:** 2025-04-08

**Authors:** Nora Jannsen, Kam C. Poon, Alexander Craze, Chang Gao, Charlotte K. Williams

**Affiliations:** ^1^ Department of Chemistry Chemistry Research Laboratory University of Oxford 12 Mansfield Road Oxford OX1 3TA UK

**Keywords:** Polymers, Epoxide and anhydride, Thermoplastic, Engineering plastics, ROCOP, Al(III) catalyst, S‐block metal catalyst

## Abstract

An organometallic Al(III)K(I) catalyst shows exceptional control in the epoxide/anhydride ring opening copolymerization (ROCOP), producing high molecular weight polyesters (*M*_n_ ∼ 100 kg·mol^−1^). The catalysis is highly effective using cyclohexene oxide, cyclopentane oxide, substituted cyclohexene oxide, and butylene oxide, each combined with phthalic anhydride. The polyesters show entanglement molecular weights, determined by oscillatory shear rheology, from 13 to 50 kg·mol^−1^ with cyclopentene and substituted cyclohexene moieties being particularly effective (highly entangled). The lead polyesters show high glass transition temperatures (94 °C < *T*_g _< 137 °C), high tensile strengths (40 MPa < *σ *< 47 MPa) and tensile modulii (0.6 GPa < *E*_y _< 0.9 GPa); their properties are similar to polystyrene. The polyesters are all recyclable by repeated cycles of compression molding, and show equivalently high thermal‐mechanical performances even over repeated recycles.

## Graphical abstract

This work exemplifies how to make high molar mass ring opening copolymerization (ROCOP) polyesters as engineering plastics, informing upon their thermal and mechanical properties. Leading polyesters, prepared by epoxide/anhydride ROCOP, show high glass transition temperatures and tensile and flexural strengths, matching or exceeding polystyrene properties.

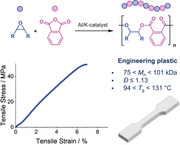

## Introduction

Polystyrene (PS) is an important thermoplastic which is manufactured at over 20 MT/annum and used in sectors including packaging, construction, electronics, and transportation.^[^ ^1^, ^2^ ^]^ The techniques employed in the commercial production of PS can be divided into two main categories: suspension processes and bulk polymerization processes.^[^ ^3^ ^]^ Depending on the type of PS, 1.9–5.8 kg of CO_2_ are emitted in order to produce one kg of PS, more than 60% of which is process fuel (1.3–4.4 kgCO_2_e·kg^−1^).^[^ ^4^ ^]^

High volumes of amorphous PS are sold; it has a glass transition temperature of ∼100 °C and high tensile strength and modulus.^[^ ^5^ ^]^ It is, however, rather brittle, but ductility can be improved using additives or copolymerization strategies.^[^ ^6^ ^]^ Polystyrene and its copolymers are difficult to recycle due to the carbon–carbon backbone and its lack of functional groups, leading to a current recycling of only 1% of PS‐waste in the US^[^ ^7^ ^]^ and around 10% in the EU.^[^ ^8^ ^]^ The low degradability of PS^[^ ^9^ ^]^ also leads to growing concern about microplastic residues.^[^ ^10^ ^]^

There is considerable interest in the replacement of pervasive hydrocarbons with more sustainable polymer alternatives. Polyesters could be attractive since the monomers may be produced from biomass/carbon dioxide, and they often show low‐energy mechanical or chemical recycling after use.^[^ ^11^, ^12^, ^13^, ^14^ ^]^ Further, polyesters can be hydrolyzed, albeit at variable rates depending on the backbone chemistry, which provides an opportunity to degrade them to metabolites like diols and diacids.^[^ ^15^, ^16^ ^]^ Currently, commercial polyesters are manufactured using step‐growth methods which occur at high temperatures. These polymerizations are not controlled, which complicates precision in chain morphology.^[^ ^17^ ^]^ Polyesters, e.g., poly(lactide) (PLA), are also produced using controlled ring‐opening polymerization, but the range of commercial cyclic esters remains very narrow.^[^ ^18^ ^]^ PLA is made from starch and is a promising sustainable replacement for fossil fuel‐derived plastics, and is widely applied in packaging and fibers.^[^ ^19^ ^]^ It has a low glass transition temperature of ∼60 °C and is brittle, which limits applications such as packaging.

An alternative controlled polymerization route to polyesters is the epoxide/anhydride ring opening copolymerization (ROCOP).^[^ ^20^, ^21^, ^22^, ^23^, ^24^, ^25^, ^26^, ^27^, ^28^, ^29^, ^30^ ^]^ It's an attractive route since there are a wide range of commercial epoxides/anhydrides, many already used in polymer manufacturing at large‐scale, and, furthermore, these monomers are also accessible from wastes or biomass.^[^ ^31^, ^32^, ^33^, ^34^ ^]^ Epoxide/anhydride ROCOP requires the use of a catalyst.^[^ ^35^ ^]^ One major issue has been to develop catalysts which are sufficiently active and tolerant (to impurities) to produce high molecular weight polyesters.^[^ ^36^ ^]^ For example, Wu and co‐workers recently compared poly(ethylene succinate) (PES) synthesized by borane/ammonium catalyzed ethylene oxide/succinic anhydride ROCOP with succinic acid and ethylene glycol polycondensation.^[^ ^37^ ^]^ Using the organocatalysis, the polycondensation route yielded polyesters with higher molecular weights (28 kg mol^−1^ vs 12 kg·mol^−1^) leading the authors to conclude that it would be preferable for the production of plastics (high *M*_n_ polymers).^[^ ^37^ ^]^ However, the selection of catalyst exerts a significant influence over the molar mass of the ROCOP polyesters and thus, there remains an opportunity to tackle limitations in molecular weight through catalyst design.

A range of different metal‐based catalysts have been successfully shown to produce ROCOP‐polyesters with molar mass, defined as M_n_> 50 kg·mol^−1^.^[^ ^38^, ^39^, ^40^, ^41^ ^]^ Coates and co‐workers pioneered in the field and prepared poly(DGA‐*alt*‐CHO) with a molar mass of *M*_n_ = 55 kg·mol^−1^ and a narrow distribution of *Ð* = 1.2, using a Zn(II) catalyst.^[^ ^41^ ^]^ Lee and co‐workers used a Co(III)‐salen catalyst to prepare poly(propylene phthalate) with *M*_n_ = 167 kg·mol^−1^ (*Ð* = 1.2).^[^ ^39^ ^]^ Fieser and team reported poly(CPMA‐*alt*‐BO) with *M*_n_ of 302 kg·mol^−1^ (*Ð* = 1.6)_,_ with a polyester selectivity of only 90%.^[^ ^40^ ^]^ In all these cases, the catalysis is complicated by chain transfer reactions with diols/diacids, which results in bimodal molecular weight distributions. Recently Wang and co‐workers presented initial mechanical tests for high molecular weight samples of poly(PA‐*alt*‐PO) and poly(PA‐*alt*‐BO) (*M*_n_ = 126–190 kg·mol^−1^, *Đ* = 1.5–1.6).^[^ ^38^ ^]^ The polyester selectivity for the samples was between 84% and 98%, and GPC analysis revealed that all samples are bimodal. The samples showed an ultimate stress of ~35 MPa and elongations at break from ‐ 11%.^[^ ^38^ ^]^

**Scheme 1:**
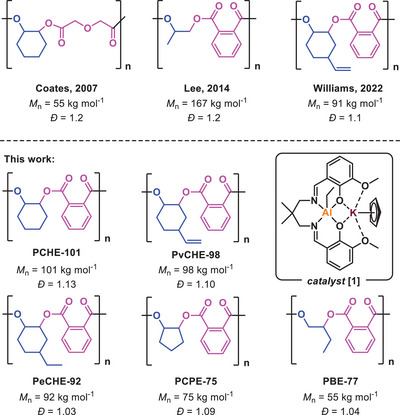
Literature examples^[^ ^38^, ^39^, ^41^ ^]^ of high molecular weight polyesters and an overview of high molecular weight polyesters in this work.

As a general strategy to produce monomodal high molecular weight polyesters, the catalyst should not feature initiating co‐ligands (e.g., halides/carboxylates) but rather ligands (e.g. alkyls/aryls) that react with diols/diacids (termed chain transfer agents) are beneficial since these allow for control.^[^ ^42^, ^43^, ^44^ ^]^ In 2022, our team reported an Al(III)K(I) organometallic catalyst which showed excellent initiation control, producing poly(PA‐*alt*‐vCHO) from vCHO/PA ROCOP with *M*_n_ = 91 kg·mol^−1^.^[^ ^43^ ^]^ Importantly, as well as accessing high molar masses, the catalyst showed monomodal molecular weight distributions, narrow dispersity, and tight initiation control. The resulting polyester was very brittle, and its tensile properties could not be tested. In developing the catalysis to make useful materials, it is important to assess the polymer's entanglement molar mass since it establishes the appropriate polymermolar mass to maximize strength and minimize processing viscosity. As part of investigating how tthe repeat unit chemistry influences such chain entanglement, four different epoxides were selected to provide chains with different backbone chemistries and substituents (Scheme 1).

## Results and Discussion

### Synthesis of High Molecular Weight Polyesters

To access a series of high molecular weight polyesters, a catalyst is required that operates effectively at low loadings, shows a broad monomer scope, and has very high degrees of initiation control. The Al/K catalyst **1** was selected because of its high activity in anhydride/epoxide ROCOP and the high crustal abundance of aluminum. **1** was synthesized in a two‐step procedure.^[^ ^43^ ^]^ The steps involve reaction of the pro‐ligand with AlEt_3_, followed by potassium coordination achieved by the addition of 1 equiv. of KCp (Figure S2).

The catalytic performance of **1** was investigated for the copolymerization of phthalic anhydride (PA) and cyclopentene oxide (CPO) or butylene oxide (BO), using a diol, benzene dimethanol (BDM), as the initiator or chain transfer agent (CTA). A catalyst:BDM:PA:epoxide ratio of 1:4:400:2000 was used initially. It showed good activities with TOF = 514 h^−1^ for PA/CPO and TOF = 224 h^−1^ for PA/BO (Table S3). The evolution of molecular weight of poly(PA‐*alt*‐CPO) was monitored during the reaction by GPC, showing a highly controlled polymerization (Figures S86 and S87). The polyester molecular weight at full anhydride conversion was *M*_n,GPC_ = 16.2 kg·mol^−1^, close to the theoretical value of *M*_n,th_ = 23.3 kg·mol^−1^, andwith a remarkably narrow dispersity *Ð* = 1.04. The molecular weight of poly(PA‐*alt*‐BO) was *M*_n,GPC_ = 20.5 kg·mol^−1^ (*Ð* = 1.05) at full conversion (*M*_n,th_ = 22.2 kg·mol^−1^). To maximize polyester molecular weights, very low catalyst loadings and high monomer purities are required. As such, PA was purified by recrystallization and four sublimations, while CPO was dried over CaH_2_ and distilled. For CPO, a series of polyesters was synthesized by varying the molar ratio of **1**:BDM:PA:CPO from 1:4:400:2000 to 1:4:3200:16 000 (Table 1). The resulting polyesters show molecular weights up to 75.1 kg·mol^−1^, with a degree of polymerization (DP) up to 324. The GPC traces (Table 1) show that all distributions are monomodal, with only the highest molecular weight polyester PCPE‐75 showing a small shoulder attributed to chains initiated from residual alcohol/acid impurities in the monomers.

**Table 1:** Synthesis of polyesters.^a)^

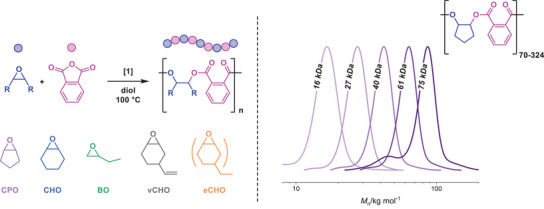							
Polymer	Epoxide	[1]:[Diol]:[PA]:[Epoxide]	Time (min)	TON^b)^ (%)	*M*_n_,_ GPC_ ^c)^ (kg·mol^−1^) [*Đ*]^d)^	*M*_n, Th_ ^e)^ (kg·mol^−1^)	DP_Exptl_ *_._* ^f)^
PCPE‐16	CPO	1:4:400:2000	65	400	16.2 [1.04]	23.3	70
PCPE‐27	CPO	1:4:800:4000	148	800	27.2 [1.04]	46.6	117
PCPE‐40	CPO	1:4:1200:6000	244	1200	40.4 [1.04]	73.1	164
PCPE‐61	CPO	1:4:1600:8000	320	1600	61.3 [1.04]	92.5	264
PCPE‐75	CPO	1:4:3200:16 000	590	3072	75.1 [1.09]	178.3	324
PCHE‐101^g)^	CHO	1:4:3200:16 000	2000	3104	101.4 [1.13]	197.4	412
PBE‐77	BO	1:4:3200:16 000	920	3200	77.4 [1.04]	176.4	352
PvCHE‐91^h)^	vCHO	1:4:1600:8000	480	1600	91.1 [1.04]	109.1	335
PvCHE‐98^h)^	vCHO	1:4:3200:16 000	450	3168	98.0 [1.10]	218.0	360
PeCHE‐92^h)^	−	−	−	−	92.1 [1.03]	−	335

^a)^ Conditions: [1]:[BDM]:[PA]:[epoxide] = 1:4:*x*:*y* where *x* is given and *x*:*y* = 1:5, *T* = 100 °C.

^b)^ Determined with ^1^H NMR spectroscopy.

^c)^ Determined by GPC in THF, at 30 °C, using narrow dispersity polystyrene standards.

^d)^ Dispersity = *M*_w_/*M*_n_, determined by GPC in THF, at 30 °C.

^e)^ Theoretical molar mass, determined by (TON · *M*_n,repeat unit_/4) + *M*_n,BDM_. (See Supporting Information for further discussion and details).

^f)^ DP, determined by *M*_n,GPC_/*M*_n,repeat unit_.

^g)^ 6 M in toluene.

^h)^ Cyclohexane diol was used instead of BDM.

Similarly, the ROCOP of PA and different epoxides, namely BO, CHO, and vCHO was performed. High molecular weight (77.4 kg·mol^−1^, *Ð =* 1.04)poly(PA‐*alt*‐BO) was isolated in good yield (80%). It was found that the ROCOP of PA and CHO at a molar ratio of 1:4:3200:16 000, in neat epoxide, leads to a highly viscous reaction mixture, resulting in insufficient mixing and in the formation of a polymer featuring ether linkages and a broad dispersity *Ð =* 2.60 (Figures S88 and S89). This viscosity issue was circumvented by the addition of minimal toluene (so as to achieve a concentration of epoxide ~ 6 M).Under these conditions the catalysis resulted in a polymer showing quantitative ester selectivity (99%) and a low dispersity, *Ð =* 1.13, as well as having the target high molecular weight *M*_n_ = 101.4 kg·mol^−1^ (Figures S11 and S17). To synthesize poly(PA‐*alt*‐vCHO), cyclohexene diol (CHD) was used as the diol (CTA) to allow for the hydrogenation of the vinyl group as post functionalization by using molecular hydrogen. The resulting polyester shows a high *M*_n_ = 98.0 kg·mol^−1^ (*Ð =* 1.10), comparable to poly(PA‐*alt*‐vCHO) already reported in the literature (91 kg·mol^−1^).^[^ ^43^ ^]^

### Hydrogenation of Poly(PA‐*alt*‐vCHO)

To easily access another new high molecularweight ROCOP polyester, poly(PA‐*alt*‐vCHO) was reduced to poly(PA‐*alt*‐eCHO). In the literature, related vinyl substituted polycarbonates were reduced to ethyl substituted polymers using *para*‐toluenesulfonyl hydrazide, but these conditions resulted in both a reduction in *M*_n_ by almost 40% and some low molar mass tailing.^[^ ^42^ ^]^ To overcome this issue, for poly(PA‐alt‐vCHO) molecular hydrogen was used for the reduction. The reaction succeeded using 10% Pd/C, at 40 bar H_2_ pressure, at 40 °C, in THF. When the aromatic diol BDM was used to make the polyester,, the subsequent hydrogenation results in the cleavage of the benzylic linkages in the diol  and results in a low‐molecular weight polyester, as evidenced by GPC (Figure S75). This problem was overcome by preparing poly(PA‐alt‐vCHO) using cyclohexane diol (CHD) instead, under the same hydrogenation conditions the target high molecular weight ethyl substituted polyester, PeCHE‐92, was prepared without compromise to either moleculare weight or dispersity (*M*_n_ = 92.1 kg·mol^−1^, *Ð* = 1.03, Figures S76 and S77).

### Thermal and Viscoelastic Properties of High Molecular Weight Polyesters

To improve polymer processability and target a range of applications, the thermal properties of polymers are important. As mentioned above, the low *T*_g_ (∼60 °C) of PLA is its main disadvantage as an engineering thermoplastic, which limits its application. The groups of Kleij and Coates pioneered the use of ROCOP for renewable aliphatic polyesters with high *T*_g_ by incorporation of highly substituted anhydrides.^[^ ^45^, ^46^, ^47^ ^]^ The glass transition temperature is dependent on the molecular weight of the material. Therefore, accessing ROCOP polymers with high *T*_g_ remains challenging, mainly because of difficulties in accessing high molecular weights. To assess the thermal properties of the new polyesters, differential scanning calorimetry (DSC), thermogravimetric analysis (TGA), dynamic mechanical thermal analysis (DMTA), and oscillatory shear rheology were used. All samples are amorphous plastics with high glass transition temperatures (*T*_g_) up to 137 °C (Table 2). The CPO‐based polyesters showed *T*_g_ values of ∼94 °C. Interestingly, the *T*_g_ of poly(PA‐*alt*‐CPO) does not increase with *M*_n_, which indicates that all samples are over the entanglement molecular weight *M*_e_ (Table S4, Figures S18–S21). Instead, the *T*_g_ is highly dependent on the ring size and substitution of the epoxide used to build the polyesters. PCHE‐101 (*T*_g_ = 137 °C), featuring the unsubstituted six‐membered ring, showed a higher *T*_g_ (Figure S25) compared to PCPE‐75 (Figure S21) which features the five‐membered ring. Due to an increased segmental motion of backbones with substituted rings compared to unsubstituted ones, the *T*_g_ of PvCHE‐98 and PeCHE‐91 are decreased to 122 °C and 127 °C, respectively. PBE‐77 shows a *T*_g,DSC_ of 48 °C, limiting its applications. The *T*_g_ values for all high molecular weight polyesters were also determined using oscillatory rheology (Figures S34–S38) and DMTA (Figures S59–S63). The decomposition temperature *T*_d,5%_ is ca. 300 °C for all high molecular weight polymers, enabling a wide processing window.

**Table 2:** Thermal and viscoelastic properties of the high molecular weight polyesters.^a)^

Polymer	*T*_g,DSC_ ^b)^ (°C)	*T*_g,rheo_ ^c)^ (°C)	*T*_g,DMA_ ^d)^ (°C)	*T*_d, 5%_ ^e)^ (°C)	*T*_t,cross_ ^f)^ (°C)	*G*‘_glass_ ^g)^ (MPa)	*τ*_t_ ^h)^ (s)	*η*_0_ ^i)^ (MPa s)	*G* ^0^ *_N_*_,rheo_ ^j)^ (MPa)	*M*_e_ ^k)^ (kg·mol^−1^)
PCPE‐75	94	112	90	293	144	1.5	0.03 ± 0.01	1.6	0.204	14 ± 2
PBE‐77	48	59	60	319	106	−	0.03 ± 0.01	7.3	0.198	13 ± 2
PCHE‐101	137	152	122	311	171	17.0	54 ± 18	2110	0.0570	50 ± 8
PvCHE‐98^l)^	122	142	117	312	196	125	26 ± 0.01	42	0.113	25 ± 4
PeCHE‐92	127	143	121	312	128	13.4	16 ± 0.5	330	0.120	24 ± 4

^a)^ TTS master curves were referenced at 170 °C, except for PBE‐77 that is referenced at 120 °C.

^b)^ Measured by DSC from the second scan at a heating rate of 10 °C·min^−1^.

^c)^ Measured by oscillatory shear rheology from the peak in the tan(*δ*) curve obtained during temperature ramp experiments at heating rates of 2 °C·min^−1^.

^d)^ Measured by DMTA from the peak in the tan(*δ*) curve obtained during temperature ramp experiments at heating rates of 3 °C·min^−1^.

^e)^ Onset of thermal decomposition, as measured by TGA.

^f)^ Terminal regime crossover temperature obtained by oscillatory shear rheology during temperature ramp experiments, 2 °C·min^−1^.

^g)^ Low‐temperature glassy plateau modulus obtained by oscillatory shear rheology during temperature ramp experiments, 2 °C·min^−1^.

^h)^ Terminal relaxation time obtained as *τ*_t_ = *ω* ^−1^ at which *G*‘ = *G*‘‘ in the TTS master curves.

^i)^ Zero shear viscosity, obtained from the low‐frequency plateau of the TTS master curves.

^j)^ Mid‐frequency rubbery plateau modulus in the TTS master curves.

^k)^ Entanglement molecular weight, as determined from the plateau modulus, *g*_N_^0^, located at the minimum of the tan(*δ*) curves in the TTS master curves.

^l)^ Sample was stabilized with 0.1 wt% of a radical inhibitor, pentaerythritol tetrakis (3,5‐di‐tert‐butyl‐4‐hydroxyhydrocinnamate) (PEHC).

The viscoelastic properties of the polymers were investigated using oscillatory rheology. Temperature ramp experiments (1% strain, 1.0 Hz, 2 °C·min^−1^) were conducted to assess the extent of chain entanglement. Except for PBE‐77, all polyesters show four characteristic viscoelastic regions: glassy, transition, rubbery plateau, and terminal regions. The rubbery plateau occurs above the *T*_g_ but when the polymer behaves like a solid because the polymer chains are still entangled. The temperature range and dynamic moduli vary with the material, and all polyesters investigated are above their entanglement molecular weight *M*_e_. The mid‐temperature rubbery plateau modulus *G* ^0^ *_N_*_,temp_ can be determined at the point where tan(*δ*) reaches its minimum. The lowest *G* ^0^ *_N_*_,temp_ was found for PCHE‐101 (*G* ^0^ *_N_*_,temp_ = 0.061 MPa) at 182 °C and the highest for PvCHE‐98 (*G* ^0^ *_N_*_,temp_ = 0.136 MPa) at 177 °C. As the temperatures are similar, the significant difference in *G* ^0^ *_N_*_,temp_ indicates a difference in entanglement between both samples, consistent with the tube model of chain entanglement.^[^ ^48^ ^]^ The glassy plateau modulus *G*’_glass_ varies significantly throughout the different polyesters from 1.5 MPa for PCPE‐75 to 125 MPa for PCHE‐101. The terminal regime crossover temperature *T*_t,cross_ occurs where *G*’ = *G*’’, following the rubbery plateau, and is dependent on the architecture of the polymer backbone and its substitution. It varies from 106 °C for PBE‐77 to 196 °C for PvCHE‐98.

**Figure 1:**
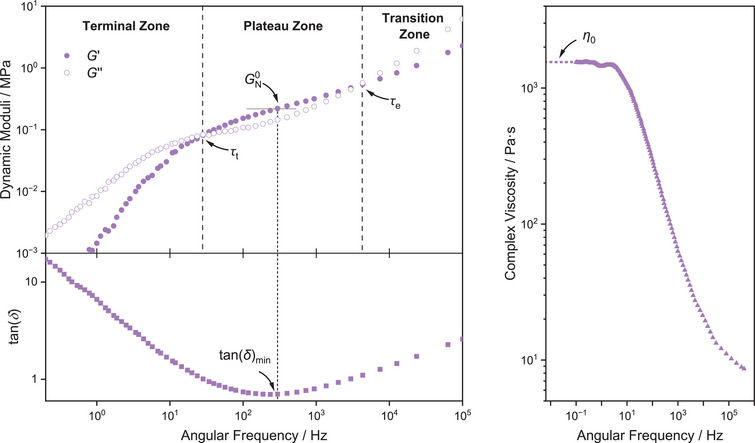
Rheological characterization of the high‐weight polyester PCPE‐75. (Left) Dynamic moduli versus frequency plot, obtained from TTS master curves. (Right) Shear viscosity versus frequency plot obtained from TTS master curves and projection of the plateau viscosity to zero‐shear.

Further insights into the molecular dynamics of the polyesters were gained by variable frequency rheology. To extend the range of the spectrum, constant 10 °C intervals were used to construct time‐temperature superposition (TTS) master curves. The reference temperature *T*_ref_ was set to 170 °C, except for PBE‐77 (*T*_ref_ = 140 °C) because of its different thermal properties. The shift factors (*a*_T_) showed good fits to the *Williams*–*Landel*–*Ferry* equation (Figures S41, S45, S49, S53, and S57). Typical thermoplastic behavior was observed for all polymers, consistent with the temperature‐ramp experiments. The polymer backbone structure has a significant influence on its viscoelastic parameters, namely on the slow and the fast relaxation processes. While the slow relaxation processes typically correspond to long‐distance chain entanglements, fast relaxation processes are attributed to short‐distance segmental rearrangements, i.e., molecular motions corresponding to the monomer repeat unit. The transition region is characterized by fast relaxation processes and gives insights into the polymer backbone effects. The frequency range of this region is different for each of the polymers in the series (Figures 1, S43, S47, S51, and S55). The shorter relaxation times for PCPE suggest that its cyclopentyl groups undergo more molecular motions than those with cyclohexyl groups. Similar short relaxation times were also observed for PBE. After moduli crossover, the rubbery plateau is reached, revealing the level of the entanglement in the samples. The storage modulus at tan(*δ*)_min_ gives the mid‐frequency rubbery plateau modulus *G* ^0^ *_N_*, which can be used to calculate the entanglement molecular weight *M*_e_ using the tube model equation (see Supporting Information).^[^ ^48^ ^]^ The *M*_e_ of the materials (see also Table 2) varies from 13 kg·mol^−1^ for PBE‐77 and 14 kg·mol^−1^ for PCPE‐75 to 50 kg·mol^−1^ for PCHE‐101. PvCHE‐89 and PeCHE‐92 show very similar *M*_e_ of 25 or 24 kg·mol^−1^, respectively. Overall, the order of *M*_e_ for all materials can be summarized as: cyclopentylene (PCPE‐75) ∼ butylene (PBE‐77) < ethyl cyclohexylene (PeCHE‐92) ∼ vinyl cyclohexylene (PvCHE‐98) < cyclohexylene (PCHE‐101). For poly(carbonates), the mechanical properties of poly(cyclohexene carbonate) were investigated by the Frey group.^[^ ^49^ ^]^ They propose that the flexible cyclohexyl group in PCHC undergoes intramolecular chair‐to‐chair conversion, thereby reducing fragility. The flipping behavior of the cyclopentyl group in the corresponding polymers has not been investigated.

The second moduli crossover results in the terminal region. At these lower frequencies, the polymers are Newtonian fluids. The terminal relaxation time *τ*_t_ = *ω* ^−1^, where *G*′ = *G*″, varies significantly throughout the polymer series. The lowest *τ*_t_ was found for PCPE‐75 and PBE‐77 (*τ*_t _= 0.03 s), which is 1800× lower than *τ*_t_(PCHE‐101) = 54 s.

The TTS‐master curves also reveal different viscous behavior of the polyesters (Figures 1, S44, S48, S52, and S56). PCHE‐101 is most viscous, 1000× times more viscous than, e.g., PCPE‐75. This result explains the need of toluene addition in the synthesis of PCHE in order to provide a homogeneous temperature distribution and to avoid the formation of ether linkages. The substitutions on the cyclohexyl ring in PvCHE and PeCHE lead to more chain movement and a lower viscosity (Table 3).

**Table 3:** Mechanical properties of polyesters: tensile data and three‐point bend DMA.

	3‐Point Bend DMA			Tensile Test			
Polymer	*σ*_f_ ^a)^ (MPa)	*ɛ*_f_ ^b)^ (%)	*E*_f_ ^c)^ (GPa)	*σ* ^d)^ (MPa)	*ɛ* ^e)^ (%)	*E*_y_ ^f)^ (GPa)	*u*_t_ ^g)^ (MJ·m^−3^)
PCPE‐75	82.0 ± 3.2	6.0 ± 0.8	3.0 ± 0.3	47.2 ± 1.4	7.2 ± 1.6	0.94 ± 0.03	2.1 ± 0.6
PvCHE‐98^h)^	89.7 ± 2.7	6.1 ± 0.5	3.1 ± 0.2	39.6 ± 2.3	8.6 ± 0.8	0.63 ± 0.04	2.0 ± 0.3
PBE‐77	61.1 ± 2.2	8.7 ± 0.8	2.9 ± 0.2	39.8 ± 3.3	7.8 ± 0.9	0.82 ± 0.09	1.9 ± 0.3
PeCHE‐92	70.2 ± 9.3	4.4 ± 0.6	2.1 ± 0.5	38.3 ± 3.3	6.9 ± 1.5	0.70 ± 0.13	1.5 ± 0.5
PCHE‐101	42.6 ± 5.8	2.2 ± 0.2	2.4 ± 0.6	32.5 ± 6.9	4.4 ± 0.7	0.93 ± 0.13	0.9 ± 0.4

^a)^ Ultimate flexural strength.

^b)^ Flexural strain at break.

^c)^ Flexural modulus, determined from the strain/stress gradient.

^d)^ Ultimate tensile strength.

^e)^ Tensile strain at break.

^f)^ Young‘s modulus, determined from the strain/stress gradient.

^g)^ Tensile toughness.

^h)^ Sample was stabilized with 0.1 wt% of a radical inhibitor, pentaerythritol tetrakis (3,5‐di‐tert‐butyl‐4‐hydroxyhydrocinnamate) (PEHC).

### Mechanical Properties

**Figure 2:**
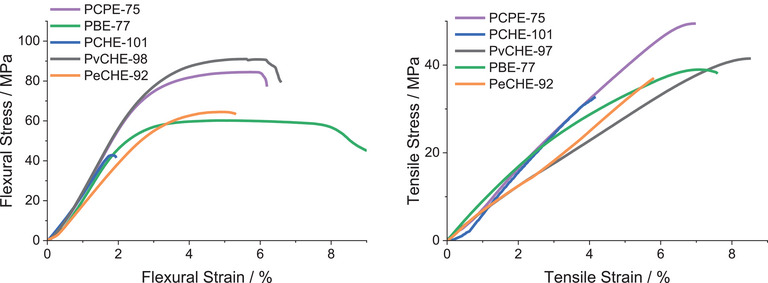
Mechanical properties of polyesters (left) three‐point bend DMA (right) tensile test.

To investigate the performance of the new polyesters, the mechanical properties were investigated. Polymer films were prepared by solvent casting into a Teflon mold and sequential compression molding. Three‐point bend DMA was performed first to compare the materials to PvCHE‐91.^[^ ^43^ ^]^ Comparing a series of different *M*_n_ values (PCPE‐27, PCPE‐40, PCPE‐61, and PCPE‐75) reveals the importance of accessing high molecular weights (Table S6). From the lowest to the highest molecular weight sample, the flexural stress and strain are significantly increased (*σ*_f_(PCPE‐27) = 16.5 MPa, *ɛ*_f_(PCPE‐27) = 0.8%; *σ*_f_(PCPE‐75) = 82.0 MPa), *ɛ*_f_(PCPE‐75) = 6.0%). PvCHE‐91^[^ ^43^ ^]^ was characterized by a three‐point bend in the literature and was slightly weaker and very brittle (*σ*_f_(PvCHE‐91) = 72.3 MPa, *ɛ*_f_(PvCHE‐91) = 3.7%).^[^ ^43^ ^]^ Interestingly, the vCHO‐based polymer PvCHE‐98 (synthesized in this work) shows improved flexural properties (Figure 2, *σ*_f_(PvCHE‐91) = 89.7 MPa, *ɛ*_f_(PvCHE‐91) = 6.1%). It displays the best flexural properties of the series. The possibility of vinyl group crosslinking, e.g., during processing, appears much less likely as GPC and NMR spectra are identical pre‐ and post‐processing (Figure S16). Despite having the highest *M*_n_, PCHE‐101 is the weakest material, as can be seen in a direct comparison with PCPE‐75. To perform these experiments, dumbbell‐shaped specimens were cut, according to ISO 527–2 type 5B. PCPE‐27 was too brittle, and its tensile properties were not tested. The tensile properties of PCPE improve with increasing molecular weight (Table S6).

Overall, the cyclohexylene sample (PCHE‐101) was weakest and most brittle, with an ultimate tensile strength of 32.5 MPa and a strain at break of 4.4% (Figure 2). The cyclopentylene sample (PCPE‐75) has the highest tensile strength (47.2 MPa) and toughness (2.1 MJ·m^−3^). The vinyl cyclohexylene sample PvCHE‐98 and the butylene sample PBE‐77 are slightly weaker but comparable to PCPE‐75.

The comparison of the mechanical properties of PCPE‐75 and PCHE‐101 (Figure 2) reveals an interesting phenomenon consistent with that reported for related polycarbonates prepared from the same epoxides:^[^ ^42^ ^]^ polyesters featuring unsubstituted cyclohexyl rings in the polymer backbone display compromised mechanical properties compared to those featuring five‐membered cyclopentyl rings. This observation can be rationalized by the high *M*_e_ of PCHE of 50 kg·mol^−1^. The molecular weight of PCHE‐101 is probably below the critical molecular weight *M*_c_, leading to its brittleness and weakness. PCPE‐75, on the other hand, is easily prepared so as to feature an *M*_n_ value which is five‐times greater than its *M*_e_ value, and thus is very likely above *M*_c_. It is assumed, that its mechanical properties would not significantly improve at even higher molecular weights. The good performance of PvCHE‐98 is also consistent with the*M*_e_ trends.

### Recycling of High Molecular Weight Polyesters

**Figure 3:**
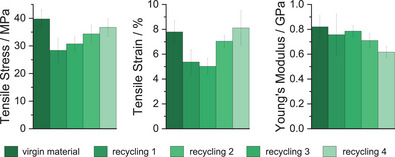
Mechanical recycling of new high‐weight polyesters: performance of mechanically recycled materials, including tensile stress, tensile strain, and Young's modulus.

To investigate the end‐of‐life recyclability of the new polyesters, PBE‐77 was mechanically recycled after mechanical testing. The tested bars were recycled by compression molding at 70 °C, followed by sequential tensile testing (Figure S78). Testing was conducted according to ISO 527, using a minimum four dumbbell‐shaped specimens, according to ISO 527–2 type 5B (Table 4, Figure 3). After the first recycle, the samples tensile stress and strain are reduced to *σ =* 28.4 MPa and *ɛ =* 5.4%. In the following cycles, *σ* and *ɛ* recover, and the last cycle shows the same mechanical properties as the virgin material. The slightly different properties in the first cycle are likely caused by defects during the processing of the polymer film that are corrected in the subsequent cycle. GPC and NMR analysis after sample recycling reveal no significant changes to the polymer structures (Figures S80–S81).

**Table 4:** Recycling and tensile test of PBE‐77.^a)^

Recycling Cycle	*σ* ^b)^ (MPa)	*ɛ* ^c)^ (%)	*E*_y_ ^d)^ (GPa)	*u*_t_ ^e)^ (MJ·m^−3^)
Virgin Material	39.8 ± 3.3	7.8 ± 0.9	0.82 ± 0.09	1.9 ± 0.3
1	28.4 ± 4.4	5.4 ± 1.0	0.76 ± 0.17	0.9 ± 0.1
2	30.7 ± 2.7	5.0 ± 0.6	0.79 ± 0.04	1.0 ± 0.1
3	34.4 ± 3.2	7.1 ± 0.5	0.71 ± 0.06	1.4 ± 0.1
4	36.7 ± 3.1	8.1 ± 1.4	0.62 ± 0.04	1.7 ± 0.4

^a)^ Conducted according to ISO 527 on a minimum of four dumbbell‐shaped specimens, according to ISO 527–2 type 5B.

^b)^ Ultimate tensile strength.

^c)^ Tensile strain at break, determined from the strain/stress gradient.

^d)^ Young‘s modulus.

^e)^ Tensile toughness.

### Commercial Comparison of High Molecular Weight Polyesters

**Figure 4:**
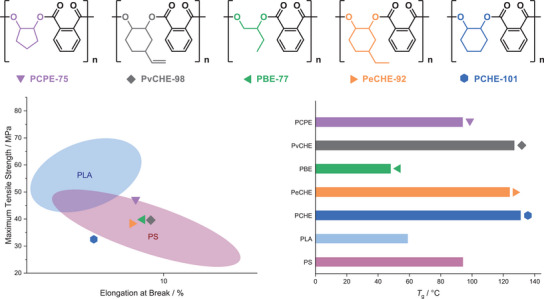
Commercial comparison of the high weight polyester series to commercial materials: a) Ashby plot, comparing the tensile strength *(σ*) and elongation (*ɛ)* at break; b) operating temperature windows.

The polyesters synthesized and tested in this work were targeted to replace current commercial plastics. In order to assess their potential as alternatives, an Ashby plot^[^ ^50^ ^]^ can be helpful (Figure 4A). The Ashby plot for known plastics polystyrene, PS, and polylactide, PLA, was created using data from different grades of commercial samples as reported in online databases (Tables S1 and S2).^[^ ^51^ ^]^ The polymer's mechanical properties are compared by plotting tensile stress versus tensile strain, showing the performance range of the commercial plastics and allowing for a direct comparison with the polyesters prepared in this work.

The worst material in the current polyesters series is PCHE‐101. Because of its brittleness and low strength, it cannot be considered a replacement for any of the commercial materials. The lead sample is PCPE‐75 since it shows a high tensile strength. Compared to PS, it is stronger and comparably brittle. PvCHE‐98 and PBE‐77 show similar mechanical properties to PCPE‐75, despite being slightly weaker and less brittle. The three materials show comparable mechanical properties to PS and may be considered as suitable replacements for it in terms of their tensile properties. Since material substitution woudl not be made only using mechanical performance data, the thermal properties of PS (*T*_g_ = 91 °C) were also compared to the plastics prepared in this series (Figure 4B). The lead cyclopentylene material shows a glass transition temperature of *T*_g_ = 94 °C, comparable to PS. The change to the cyclohexyl featuring backbone improves the high temperature stability even more since it features a *T*_g_ = 137 °C, and the ethyl and vinyl substitution of the cyclohexylenering leads to similar high temperature stabilities (*T*_g_ = 127° or 122 °C). ROCOP polyesters featuring such high *T*_g_ values are still rather rare in the literature and all these values are higher than the glass transition temperatures reported for the analogous ROCOP polycarbonates.^[^ ^42^ ^]^ PBE‐77 represents an exception within the series, since its aliphatic polyester structure results in a lower glass transition temperature of 48 °C. The viscoelastic properties of commercial PS (*M*_w_ = 38 kg·mol^−1^ and *M*_w_ = 280 kg·mol^−1^) were also investigated using oscillatory rheology. Temperature ramp experiments (1% strain, 1.0 Hz, 2 °C·min^−1^) reveal that its complex viscosity increases with the molecular weight of the PS sample (Figures S83 and S84). The complex viscosity of PCPE‐75 was found to sit between the values of the two PS samples (Figure S85). These experiments suggest that PCPE‐75 may warrant further exploration as a replacement for PS, as it seems to match some aspects of its thermal‐mechanical and rheological properties.

PLA is a bioderived plastic that is both strong (*σ* = 44–68 MPa) but brittle (*ɛ* = 3.0%–7.5%), it is already used in many applications. Comparing PLA and the lead plastic from the series PCPE‐75 (Figure 4A), reveals that they both show similar strengths and elongations at break. Applications of PLA may be more limited by its low glass transition temperature (∼60 °C). In comparison, PCPE‐75 (*T*_g_ = 94 °C) could be an interesting material for higher temperature applications, where PLA cannot be used.

## Conclusion

An organometallic Al(III)K(I) catalyst was applied to synthesize a series of high molecular weight polyesters, *M*_n_ = 75–101 kg·mol^−1^, by copolymerization of phthalic anhydride (PA) with epoxides including vinyl‐cyclohexene oxide (vCHO), cyclohexene oxide (CHO), cyclopentene oxide (CPO), or butylene oxide (BO). The new materials, especially poly(PA‐*alt*‐CPO), show significant potential as future thermoplastics. Poly(PA‐*alt*‐CPO) showed the best mechanical properties due to its low *M*_e_ (14 kg·mol^−1^). It had high tensile (47 MPa) and flexural strengths (82 MPa), moderate elongation at break (7%), high tensile toughness (2 MJ·m^−3^), a high glass transition temperature (*T*_g_ = 90 °C, 94 °C, or 112 °C by DMA, DSC, or rheology), and a low zero shear viscosity (0.0016 MPa·s). Poly(PA‐*alt*‐CPO) features a five‐membered cyclopentylene ring and features a significantly lower entanglement molecular weight compared to the equivalent polymer featuring a six‐membered cyclohexylene ring poly(PA‐*alt*‐CHO) (*M*_e_ = 50 kg·mol^−1^). One of the lead materials, poly(PA‐*alt*‐BO), was mechanically recycled four times, showing no changes to its tensile properties or structure, as confirmed by NMR and GPC analysis. The polyesters outperform some current grades of polystyrene in terms of both tensile and thermal properties and, due to their high *T*_g_ values may be useful for high‐temperature applications where polylactide is ineffective.

## Supporting Information

Additional supporting information can be found online in the Supporting Information section at the end of this article. The authors have cited additional references within the Supporting Information.^[^ ^52^, ^53^ ^]^

## Conflict of Interests

The authors declare no conflict of interest.

## Supporting information



Supporting Information

## Data Availability

The data that support the findings of this study are available in the Supporting Information of this article.
