# Supplementary material for: Controlled Catalysis Delivering High Molecular Weight Polyesters as Recyclable Alternatives to Polystyrenes
Source: Angew Chem Int Ed Engl. 2025 Apr 8;64(23):e202505070. doi: 10.1002/anie.202505070 (PMC12124439; doi:10.1002/anie.202505070)
Supplement: Supplementary file 1 — Supporting Information [file ANIE-64-e202505070-s001.docx]

Supporting Information:

**Controlled Catalysis Delivering High Molecular Weight Polyesters as Recyclable Alternatives to Polystyrenes**

Dr. Nora Jannsen, Dr. Kam C. Poon, Alexander Craze, Chang Gao, and Prof. Charlotte K. Williams*

Chemistry Research Laboratory, Department of Chemistry, University of Oxford, Oxford, OX1 3TA, U.K.

E-mail: [charlotte.williams@chem.ox.ac.uk](mailto:charlotte.williams@chem.ox.ac.uk)

Contents

[Figures 3](#_Toc193386322)

[General Procedures and Materials 7](#_Toc193386323)

[Methods 7](#_Toc193386324)

[Experimental Details 9](#_Toc193386325)

[Complex synthesis 9](#_Toc193386326)

[Synthesis of L_van_Al(Et) 9](#_Toc193386327)

[Synthesis of L_van_Al(Et)K(Cp) 10](#_Toc193386328)

[Polymer synthesis 11](#_Toc193386329)

[General procedure 11](#_Toc193386330)

[Synthesis of PCPE-75 11](#_Toc193386331)

[Synthesis of PBE-77 11](#_Toc193386332)

[Synthesis of PvCHE-98 12](#_Toc193386333)

[Synthesis of PCHE-101 12](#_Toc193386334)

[NMR spectra of high molecular weight polyesters 13](#_Toc193386335)

[GPC traces of high molecular weight polyesters 18](#_Toc193386336)

[Differential Scanning Calorimetry (DSC) 20](#_Toc193386337)

[Thermal Gravimetric Analysis (TGA) 24](#_Toc193386338)

[Rheology 28](#_Toc193386339)

[Temperature ramp experiments 28](#_Toc193386340)

[Temperature Time Superposition (TTS) experiments 33](#_Toc193386341)

[Temperature Time Superposition (TTS) for PCPE-75 33](#_Toc193386342)

[Temperature Time Superposition (TTS) for PBE-77 35](#_Toc193386343)

[Temperature Time Superposition (TTS) for PeCHE-92 37](#_Toc193386344)

[Temperature Time Superposition (TTS) for PvCHE-98 39](#_Toc193386345)

[Temperature Time Superposition (TTS) for PCHE-101 41](#_Toc193386346)

[Dynamic Mechanical Analyzer (DMA) 43](#_Toc193386347)

[Dynamic Mechanical Thermal Analysis (DMTA) 43](#_Toc193386348)

[3-Point bend experiments 47](#_Toc193386349)

[Tensile Test 51](#_Toc193386350)

[Hydrogenation of PvCHE-91 53](#_Toc193386351)

[Recycling Study 55](#_Toc193386352)

[Procedure 55](#_Toc193386353)

[Tensile Test of Recycled Polymer 56](#_Toc193386354)

[GPC of Recycled Polymer 57](#_Toc193386355)

[NMR of Recycled Polymer 57](#_Toc193386356)

[Commercial comparison 58](#_Toc193386357)

[Ashby Plot 58](#_Toc193386358)

[Rheology 60](#_Toc193386359)

[Additional Tables 63](#_Toc193386360)

[Additional Figures 65](#_Toc193386361)

[References 67](#_Toc193386362)

# Figures

[Figure S 1. ^1^H NMR spectrum (CDCl_3_, 400 MHz, 298 K) of L_van_Al(Et). 9](#_Toc193702465)

[Figure S 2. ^1^H NMR spectrum (THF-*d*_8_, 400 MHz, 298 K) of L_van_Al(Et)K(Cp). 10](#_Toc193702466)

[Figure S 3. ^1^H NMR spectrum (CDCl_3_, 400 MHz, 298 K) of PCPE-75. 13](#_Toc193702467)

[Figure S 4. ^1^H COSY NMR spectrum (CDCl_3_, 400 MHz, 298 K) of PCPE-75. 13](#_Toc193702468)

[Figure S 5. ^1^H NMR spectrum (CDCl_3_, 400 MHz, 298 K) of PBE-77. 14](#_Toc193702469)

[Figure S 6. ^1^H COSY NMR spectrum (CDCl_3_, 400 MHz, 298 K) of PBE-77. 14](#_Toc193702470)

[Figure S 7. ^1^H NMR spectrum (CDCl_3_, 400 MHz, 298 K) of PeCHE-92. 15](#_Toc193702471)

[Figure S 8. ^1^H COSY NMR spectrum (CDCl_3_, 400 MHz, 298 K) of PeCHE-92. 15](#_Toc193702472)

[Figure S 9. ^1^H NMR spectrum (CDCl_3_, 400 MHz, 298 K) of PvCHE-98 16](#_Toc193702473)

[Figure S 10. ^1^H COSY NMR spectrum (CDCl_3_, 400 MHz, 298 K) of PvCHE-98 16](#_Toc193702474)

[Figure S 11. ^1^H NMR spectrum (CDCl_3,_ 400 MHz, 298 K) of PCHE-101. 17](#_Toc193702475)

[Figure S 12. ^1^H COSY NMR spectrum (CDCl_3_, 400 MHz, 298 K) of PCHE-101. 17](#_Toc193702476)

[Figure S 13. GPC trace (THF, 303 K) of PCPE-75. 18](#_Toc193702477)

[Figure S 14. GPC trace (THF, 303 K) of PBE-77. 18](#_Toc193702478)

[Figure S 15. GPC trace (THF, 303 K) of PeCHE-92. 18](#_Toc193702479)

[Figure S 16. GPC trace (THF, 303 K) of PvCHE-98 before (black line) and after (red dots) processing. 19](#_Toc193702480)

[Figure S 17. GPC trace (THF, 303 K) of PCHE-101. 19](#_Toc193702481)

[Figure S 18. Differential Scanning Calorimetry (DSC) data for PCPE-27. 20](#_Toc193702482)

[Figure S 19. Differential Scanning Calorimetry (DSC) data for PCPE-40. 20](#_Toc193702483)

[Figure S 20. Differential Scanning Calorimetry (DSC) data for PCPE-61. 21](#_Toc193702484)

[Figure S 21. Differential Scanning Calorimetry (DSC) data for PCPE-75. 21](#_Toc193702485)

[Figure S 22. Differential Scanning Calorimetry (DSC) data for PBE-77. 22](#_Toc193702486)

[Figure S 23. Differential Scanning Calorimetry (DSC) data for PeCHE-92. 22](#_Toc193702487)

[Figure S 24. Differential Scanning Calorimetry (DSC) data for PvCHE-98, stabilized with 0.1 wt % of a radical inhibitor, pentaerythritol tetrakis (3,5-di-tert-butyl-4-hydroxyhydrocinnamate) (PEHC). 23](#_Toc193702488)

[Figure S 25. Differential Scanning Calorimetry (DSC) data for PCHE-101. 23](#_Toc193702489)

[Figure S 26. Thermogravimetric Analysis (TGA) data for PCPE-27. 24](#_Toc193702490)

[Figure S 27. Thermogravimetric Analysis (TGA) data for PCPE-40. 24](#_Toc193702491)

[Figure S 28. Thermogravimetric Analysis (TGA) data for PCPE-61. 25](#_Toc193702492)

[Figure S 29. Thermogravimetric Analysis (TGA) data for PCPE-75. 25](#_Toc193702493)

[Figure S 30. Thermogravimetric Analysis (TGA) data for PBE-77. 26](#_Toc193702494)

[Figure S 31. Thermogravimetric Analysis (TGA) data for PeCHE-92. 26](#_Toc193702495)

[Figure S 32. Thermogravimetric Analysis (TGA) data for PvCHE-98, stabilized with 0.1 wt % of a radical inhibitor, pentaerythritol tetrakis (3,5-di-tert-butyl-4-hydroxyhydrocinnamate) (PEHC). 27](#_Toc193702496)

[Figure S 33. Thermogravimetric Analysis (TGA) data for PCHE-101. 27](#_Toc193702497)

[Figure S 34. Oscillatory temperature ramp of PCPE-75 (1% strain, 1.0 Hz, 2 °C·min^-1^) between 100-160 °C. Top: dynamic moduli, *G*’ and *G*‘‘; bottom: tan(*δ*). 28](#_Toc193702498)

[Figure S 35. Oscillatory temperature ramp of PBE-77 (1% strain, 1.0 Hz, 2 °C·min^-1^) between 50-140 °C. Top: dynamic moduli, *G*’ and *G*‘‘; bottom: tan(*δ*). 29](#_Toc193702499)

[Figure S 36. Oscillatory temperature ramp of PeCHE-92 (1% strain, 1.0 Hz, 2 °C·min^-1^) between 110-210 °C. Top: dynamic moduli, *G*’ and *G*‘‘; bottom: tan(*δ*). 30](#_Toc193702500)

[Figure S 37. Oscillatory temperature ramp of PvCHE-98, stabilized with 0.1 wt % of a radical inhibitor, pentaerythritol tetrakis (3,5-di-tert-butyl-4-hydroxyhydrocinnamate) (PEHC). (1% strain, 1.0 Hz, 2 °C·min^-1^) between 120-210 °C. Top: dynamic moduli, *G*’ and *G*‘‘; bottom: tan(*δ*). 31](#_Toc193702501)

[Figure S 38. Oscillatory temperature ramp of PCHE-101 (1% strain, 1.0 Hz, 2 °C·min^-1^) between 120-210 °C. Top: dynamic moduli, *G*’ and *G*‘‘; bottom: tan(*δ*). 32](#_Toc193702502)

[Figure S 39. Mastercurves of PCPE-75 constructed by time-temperature superposition (TTS) referenced to 170 °C (1% strain, 0.01-100 Hz). Top: dynamic moduli, *G*’ and *G*’’; bottom: tan(*δ*). 33](#_Toc193702503)

[Figure S 40. Mastercurves of PCPE-75 constructed by time-temperature superposition (TTS) referenced to 170 °C (1% strain, 0.01-100 Hz): complex viscosity, *η**. 33](#_Toc193702504)

[Figure S 41. Oscillatory frequency sweeps (1% strain) of PCPE-75 employed in the creation of mastercurves. 34](#_Toc193702505)

[Figure S 42. Temperature dependence of the shift factors, αT, employed for the construction of the mastercurve of PCPE-75 (black diamonds) referenced at 170 °C and fitted curve (red line) to the WLF equation. 34](#_Toc193702506)

[Figure S 43. Mastercurves of PBE-77 constructed by time-temperature superposition (TTS) referenced to 140 °C (1% strain, 0.01-100 Hz). Top: dynamic moduli, *G*’ and *G*’’; bottom: tan(*δ*). 35](#_Toc193702507)

[Figure S 44. Mastercurves of PBE-77 constructed by time-temperature superposition (TTS) referenced to 170 °C (1% strain, 0.01-100 Hz): complex viscosity, *η**. 35](#_Toc193702508)

[Figure S 45. Oscillatory frequency sweeps (1% strain) of PBE-77 employed in the creation of mastercurves. 36](#_Toc193702509)

[Figure S 46. Temperature dependence of the shift factors, αT, employed for the construction of the mastercurve of PBE-77 (black diamonds) referenced at 140 °C and fitted curve (red line) to the WLF equation. 36](#_Toc193702510)

[Figure S 47. Mastercurves of PeCHE-92 constructed by time-temperature superposition (TTS) referenced to 170 °C (1% strain, 0.01-100 Hz). Top: dynamic moduli, *G*’ and *G*’’; bottom: tan(*δ*). 37](#_Toc193702511)

[Figure S 48. Mastercurves of PeCHE-92 constructed by time-temperature superposition (TTS) referenced to 170 °C (1% strain, 0.01-100 Hz): complex viscosity, *η**. 37](#_Toc193702512)

[Figure S 49. Oscillatory frequency sweeps (1% strain) of PeCHE-92 employed in the creation of mastercurves. 38](#_Toc193702513)

[Figure S 50. Temperature dependence of the shift factors, αT, employed for the construction of the mastercurve of PeCHE-92 (black diamonds) referenced at 170 °C and fitted curve (red line) to the WLF equation. 38](#_Toc193702514)

[Figure S 51. Mastercurves of PvCHE-98 (stabilized with 0.1 wt % of a radical inhibitor, pentaerythritol tetrakis (3,5-di-tert-butyl-4-hydroxyhydrocinnamate) (PEHC)) constructed by time-temperature superposition (TTS) referenced to 170 °C (1% strain, 0.01-100 Hz). Top: dynamic moduli, *G*’ and *G*’’; bottom: tan(*δ*). 39](#_Toc193702515)

[Figure S 52. Mastercurves of PvCHE-98 constructed by time-temperature superposition (TTS) referenced to 170 °C (1% strain, 0.01-100 Hz): complex viscosity, *η**. 39](#_Toc193702516)

[Figure S 53. Oscillatory frequency sweeps (1% strain) of PvCHE-98 employed in the creation of mastercurves. 40](#_Toc193702517)

[Figure S 54. Temperature dependence of the shift factors, αT, employed for the construction of the mastercurve of PvCHE-98 (black diamonds) referenced at 170 °C and fitted curve (red line) to the WLF equation. 40](#_Toc193702518)

[Figure S 55. Mastercurves of PCHE-101 constructed by time-temperature superposition (TTS) referenced to 170 °C (1% strain, 0.01-100 Hz). Top: dynamic moduli, *G*’ and *G*’’; bottom: tan(*δ*). 41](#_Toc193702519)

[Figure S 56. Mastercurves of PCHE-101 constructed by time-temperature superposition (TTS) referenced to 170 °C (1% strain, 0.01-100 Hz): complex viscosity, *η**. 41](#_Toc193702520)

[Figure S 57. Oscillatory frequency sweeps (1% strain) of PCHE-101 employed in the creation of mastercurves. 42](#_Toc193702521)

[Figure S 58. Temperature dependence of the shift factors, αT, employed for the construction of the mastercurve of PCHE-101 (black diamonds) referenced at 170 °C and fitted curve (red line) to the WLF equation. 42](#_Toc193702522)

[Figure S 59. Dynamic mechanical temperature analysis (DMTA) temperature sweeps for PCPE-75. 43](#_Toc193702523)

[Figure S 60. Dynamic mechanical temperature analysis (DMTA) temperature sweeps for PBE-77. 44](#_Toc193702524)

[Figure S 61. Dynamic mechanical temperature analysis (DMTA) temperature sweeps for PeCHE-92. 45](#_Toc193702525)

[Figure S 62. Dynamic mechanical temperature analysis (DMTA) temperature sweeps for PvCHE-98, stabilized with 0.1 wt % of a radical inhibitor, pentaerythritol tetrakis (3,5-di-tert-butyl-4-hydroxyhydrocinnamate) (PEHC). 46](#_Toc193702526)

[Figure S 63. Dynamic mechanical temperature analysis (DMTA) temperature sweeps for PCHE-101. 47](#_Toc193702527)

[Figure S 64. 3-Point bend dynamic mechanical analysis (DMA) data for PCPE-27. 48](#_Toc193702528)

[Figure S 65. 3-Point bend dynamic mechanical analysis (DMA) data for PCPE-40. 48](#_Toc193702529)

[Figure S 66. 3-Point bend dynamic mechanical analysis (DMA) data for PCPE-75. 49](#_Toc193702530)

[Figure S 67. 3-Point bend dynamic mechanical analysis (DMA) data for PBE-77. 49](#_Toc193702531)

[Figure S 68. 3-Point bend dynamic mechanical analysis (DMA) data for PeCHE-92. 50](#_Toc193702532)

[Figure S 69. 3-Point bend dynamic mechanical analysis (DMA) data for PvCHE-98, stabilized with 0.1 wt % of a radical inhibitor, pentaerythritol tetrakis (3,5-di-tert-butyl-4-hydroxyhydrocinnamate) (PEHC). 50](#_Toc193702533)

[Figure S 70. 3-Point bend dynamic mechanical analysis (DMA) data for PCHE-101. 51](#_Toc193702534)

[Figure S 71. Tensile strain-stress plots for PCPE-75. 52](#_Toc193702535)

[Figure S 72. Tensile strain-stress plots for PBE-77. 52](#_Toc193702536)

[Figure S 73. Tensile strain-stress plots for PvCHE-98, stabilized with 0.1 wt % of a radical inhibitor, pentaerythritol tetrakis (3,5-di-tert-butyl-4-hydroxyhydrocinnamate) (PEHC). 53](#_Toc193702537)

[Figure S 74. Tensile strain-stress plots for PCHE-101. 53](#_Toc193702538)

[Figure S 75. GPC trace (THF, 303 K) for BDM-initiated PvCHE-75, before (dark grey) and after (red) hydrogenation (in THF, 40 bar H_2_, 40 °C). 54](#_Toc193702539)

[Figure S 76. ^1^H NMR spectrum (CDCl_3_, 298 K) for CHD-initiated PvCHE-91, before (bottom) and after (top) hydrogenation (in THF, 40 bar H_2_, 40 °C). 54](#_Toc193702540)

[Figure S 77. GPC trace (THF, 303 K) for CHD-initiated PvCHE-92, before (dark grey) and after (orange) hydrogenation (in THF, 40 bar H_2_, 40 °C). 55](#_Toc193702541)

[Figure S 78. Pictures of mechanical recycling. 56](#_Toc193702542)

[Figure S 79. Tensile strain-stress plots for the recycling of PBE-77. A) first recycling, B) second recycling, C) third recycling, D) fourth recycling. 57](#_Toc193702543)

[Figure S 80. GPC trace (THF, 303 K) for PBE-77, before (dark grey) and after (red) recycling (compression moulding at 70 °C, 1 h). 58](#_Toc193702544)

[Figure S 81. ^1^H NMR spectrum (CDCl_3_, 298 K) for PBE-77, before (bottom) and after (top) recycling (compression moulding at 70 °C, 1 h). 58](#_Toc193702545)

[Figure S 82. Ashby plot from data for different grades of PS and PLA from online databases.^[4]^ 60](#_Toc193702546)

[Figure S 83. Oscillatory temperature ramp of PS-38 (1% strain, 1.0 Hz, 2 °C·min^-1^) between 120-210 °C. Top: dynamic moduli, *G*’ and *G*‘‘; bottom: tan(*δ*). 61](#_Toc193702547)

[Figure S 84. Oscillatory temperature ramp of PS-280 (1% strain, 1.0 Hz, 2 °C·min^-1^) between 120-210 °C. Top: dynamic moduli, *G*’ and *G*‘‘; bottom: tan(*δ*). 62](#_Toc193702548)

[Figure S 85. Complex viscosity from oscillatory temperature ramp of PS-38, PS280 and PCPE-75 (1% strain, 1.0 Hz, 2 °C·min^-1^) between 65-220 °C. 63](#_Toc193702549)

[Figure S 86. GPC traces (THF, 303 K) for poly(PA-*alt*-CPO) during the reaction. 66](#_Toc193702550)

[Figure S 87. *M*_n_ vs. conversion plot for poly(PA-*alt*-CPO). 66](#_Toc193702551)

[Figure S 88. ^1^H NMR spectrum (CDCl_3_, 298 K) for the ring-opening copolymerization of CHO and PA with L_van_Al(Et)K(Cp) and BDM (cat:BDM:PA:CHO = 1:4:3200:16000), performed in neat CHO. 67](#_Toc193702552)

[Figure S 89. GPC trace (THF, 303 K) for poly(PA-*alt*-CHO), polymerization performed in neat CHO. 67](#_Toc193702553)

[Figure S 90. ^1^H NMR spectrum (CDCl_3,_ 400 MHz, 298 K) of PCHE-101: integration of the ether region. 67](#_Toc193702554)

# General Procedures and Materials

All synthetic manipulations were carried out in a nitrogen filled glovebox, or a dual manifold nitrogen-vacuum Schlenk line. Solvents used in synthesis were collected from a solvent purification system (SPS), degassed with three freeze-pump-thaw cycles and stored over 3 Å molecular sieves under an inert atmosphere, unless otherwise stated. All materials were stored under a nitrogen atmosphere, in a glovebox. Triethyl aluminium (93%) was purchased from Sigma Aldrich and was used as received. Vinyl cyclohexene oxide (vCHO, Sigma Aldrich) and cyclopentene oxide (CPO, Sigma Aldrich) were purified by stirring over calcium hydride, followed by fractional distillation. Cyclohexene oxide (CHO, Acros Organics) and butylene oxide (BO, Sigma Aldrich) were purified by a three-fold distillation process. Firstly, the epoxide was stirred over calcium hydride for 16 hours and subsequently fractionally distilled. The distillate was then stirred with *n*BuLi in hexanes (5 v/v%) for 16 hours, and the mixture was subsequently fractionally distilled. A final, subsequent, distillation of the collected fraction was performed. Phthalic anhydride (PA, Sigma Aldrich) was purified though a three-step procedure. Firstly, PA was stirred in dry toluene at room temperature for 16 hours. The supernatant was filtered, and the toluene subsequently removed in vacuo. The resultant white powder recrystallised from hot (60 °C) chloroform and subsequently sublimed four times under vacuum at 80 °C. Polystyrene (PS-38, PS-280, Sigma Aldrich) were used without purification.

The pro-ligand, L_van_H_2_,^[1]^ and the complexes L_van_Al(Et),^[2]^ and L_van_Al(Et)K(Cp)^[3]^ were synthesised following literature procedures.

# Methods

**NMR Spectroscopy:** ^1^H NMR spectra were obtained using a Bruker Avance III HD 400 NMR spectrometer. ^13^C{^1^H} NMR were obtained with either Bruker AV III HD 500 MHz NMR, Bruker NEO 600 MHz with broadband helium cryoprobe spectrometers.

**GPC analysis:** Carried out using a Shimadzu LC-20AD instrument, equipped with a Refractive Index (RI) detector and two PSS SDV 5 µm linear M columns. The eluent used was HPLC-grade THF, heated to 30 °C, and with a flow rate of 1.0 mL min^-1^.

**Differential Scanning Calorimetry (DSC):** Carried out using a TA discovery 25 auto. Samples were heated to 200 °C for 5 minutes to remove thermal history, before heating and cooling from 30 °C to 200 °C, at a rate of 10 °C min^-1^. Glass transition temperatures (*T*_g_) were recorded from the midpoint of the transition during the third heating curve.

**Thermal Gravimetric Analysis (TGA):** Carried out using a TGA5500 System (TA Instruments), equipped with the TRIOS software package. Samples were heated from 30 °C to 500 °C, under continuous N_2_ flow, at a rate of 20 °C min^-1^.

**Rheology:** Oscillatory shear measurements were performed on an ARES-G2 rheometer (TA Instruments) between two stainless steel 8 mm parallel plates. Clear round disks 8 mm in diameter, were punch-cut with a hole borer from compression moulded homogeneous films (0.3–0.8 mm thickness). The specimen disks were loaded above the *T*_g_ of the polyesters, under a stream of N_2_, inside the rheometer furnace. The axial force was allowed to freely relax with a the geometry gap to 300-800 μm. Dynamic tests were performed in the viscoelastic linear region after performing dynamic strain sweeps. Oscillatory temperature ramps (1% strain, 1.0 Hz, 2 °C · min^-1^) were performed on cooling from the maximum running temperature. Consecutive oscillatory frequency sweeps (1% strain, 0.01–100 Hz) were performed at 10 °C intervals, with 1 min equilibration time between runs. The data was successfully combined into the corresponding time-temperature superposition (TTS) mastercurves with the TRIOS Software Version 4.2.1 (TA Instruments), and referenced to 170 °C for all samples except PBE, where 140 °C was used.

**Dynamic Mechanical Analysis (DMA):** Carried out on a DMA 850 (TA Instruments). For 3-point bend experiments the following procedure was applied: samples were subjected to a strain ramp, at room temperature, from 0% to break at 0.1% strain min^-1^.

**Dynamic Mechanical Analysis (DMTA):** Thermal analysis (DMTA) was carried out using a DMA850 (TA Instruments), using an ACS III cooling system. Specimens of uniform width (5.3 mm) were cut using two parallel blades. Samples were heated from -80 °C to 200 °C (or until the material deformed beyond the limits of the geometry employed), at a rate of 3 °C min^‑1^, with a frequency of 1 Hz, 0.1 N pre-load force and 0.1% strain amplitude.

**Compression Moulding:** Polymer films were processed or reprocessed using the Carver mini CH CE Press (5420CE.4010C00) with heated plates and a hydraulic press. Pressing temperatures: *T*_press_(PCPE) = 120 °C, *T*_press_(PBE) = 80 °C, *T*_press_(PeCHE) = 150 °C, *T*_press_(PvCHE) = 150 °C, *T*_press_(PCHE) = 160 °C.

**Tensile Testing:** Carried out using an Instron 8600 series universal testing system using a 50 N load cell and 250 N pneumatic grips. Dumbbell-shaped specimens were cut using a Zwick ZCP020 cutting press equipped with a cutting device for ISO 527-2 type 5B. Uniaxial extension experiments (10 mm min^-1^ cross-head speed) were run according to ISO 527.

# Experimental Details

## Complex synthesis

### Synthesis of L_van_Al(Et)

The complex was synthesized follwing an adapted literature procedure.^[2-3]^ The ligand L_van_H_2_ (518 mg, 1.4 mmol) was dissolved in 10 mL anhydrous toluene. AlEt_3_ (180 mg, 1.6 mmol) was added to the yellow solution at -30 °C. The reaction mixture was stirred over night at room temperature leading to precipitation of a light yellow powder. The solution was removed with a cannular and the powder was washed with tolunene (2 x 10 mL) and subsequently dried in vacuum. The spectra of the complex were consistent with those previously reported.

Figure S 1. ^1^H NMR spectrum (CDCl_3_, 400 MHz, 298 K) of L_van_Al(Et).

### Synthesis of L_van_Al(Et)K(Cp)

Synthesis of L_van_Al(Et)K(Cp) was synthesized die to the litertature.^[3]^ L_van_Al(Et) (64.4 mg, 0.15 mmol) was dissolved in 5 mL anhydrous THF and mixed with KCp (15.9 mg, 0.15 mmol) in 5 mL anhydrous THF. The solution was stirred at room temperature for 30 min and subsequently dried in vacuum. The spectra of the complex were consistent with those previously reported.

Figure S 2. ^1^H NMR spectrum (THF-*d*_8_, 400 MHz, 298 K) of L_van_Al(Et)K(Cp).

## Polymer synthesis

### General procedure

L_van_Al(Et)K(Cp), 1,4-benzenedimethanol (1,4-BDM) or 1,2-cyclohenxenediol (1,2-CHD), and the stated epoxide and anhydride monomers were added to a dried vial or Schlenk, equipped with a magnetic stirrer bar, in the ratios specified ([1]:[diol]:[PA]:[epoxide] = 1:4:400:2000 - 1:4:3200:16000). The vial was sealed with a melamine-cap containing a Teflon inlay, and further sealed with electrical insulation tape. This sealed vial or Schlenk was heated to 100 °C for the stated time. Aliquoting was performed by setting up several vials and stopping the polymerization after different reaction times. The polymerizations were quenched by exposing the reaction mixture to air, followed by removal of volatiles. Polymers were purified by precipitation from DCM/MeOH and dried under vacuum at 40 °C.

### Synthesis of PCPE-75

PA (890 mg, 6 mmol), L_van_Al(Et)K(Cp) (1 mg, 1.9·10^-3^ mmol) and 1,4-BDM (1 mg, 7.5·10^‑3^ mmol), were added to a dried vial and dissolved in CPO (2.5 mL, 30 mmol). Stock solutions in CPO were freshly prepared and used for L_van_Al(Et)K(Cp) (0.013 M) and 1,4-BDM (0.23 M). The vial was sealed with a melamine-cap containing a Teflon inlay, and further sealed with electrical insulation tape. This sealed vial was heated to 100 °C for 10 h. The polymerization was quenched by exposing the reaction mixture to air. PCPE-75 was purified by precipitation from DCM/MeOH (3x) and dried under vacuum at 40 °C. 1.2 g of the polymer was isolated (yield = 86%).

### Synthesis of PBE-77

PA (1023 mg, 6.9 mmol), L_van_Al(Et)K(Cp) (1.2 mg, 2.2·10^-3^ mmol) and 1,4-BDM (1.2 mg, 8.7·10^‑3^ mmol), were added to a dried vial and dissolved in BO (3 mL, 34 mmol). Stock solutions in BO were freshly prepared and used for L_van_Al(Et)K(Cp) (0.02 M) and 1,4-BDM (0.23 M). The vial was sealed with a melamine-cap containing a Teflon inlay, and further sealed with electrical insulation tape. This sealed vial was heated to 100 °C for 15 h. The polymerization was quenched by exposing the reaction mixture to air. PBE-77 was purified by precipitation from DCM/MeOH (3x) and dried under vacuum at 40 °C. 1.2 g of the polymer was isolated (yield = 79%).

### Synthesis of PvCHE-98

PA (910 mg, 6.1 mmol), L_van_Al(Et)K(Cp) (1.1 mg, 1.9·10^-3^ mmol) and 1,2-CHD (0.9 mg, 7.6·10^‑3^ mmol), were added to a dried Schlenk and dissolved in vCHO (4 mL, 31 mmol). Stock solutions in vCHO were freshly prepared and used for L_van_Al(Et)K(Cp) (0.02 M) and 1,2-CHD (0.15 M). This sealed Schlenk was heated to 100 °C for 7 h. The polymerization was quenched by exposing the reaction mixture to air. PvCHE-98 was purified by precipitation from DCM/MeOH (3x) and dried under vacuum at 40 °C. 1.1 g of the polymer was isolated (yield = 67%).

### Synthesis of PCHE-101

PA (873.8 mg, 5.9 mmol), L_van_Al(Et)K(Cp) (1.1 mg, 1.9·10^-3^ mmol) and 1,4-BDM (1.0 mg, 7.4·10^‑3^ mmol), were added to a dried Schlenk and dissolved in CHO (3 mL, 30 mmol) and 5 mL toluene. Stock solutions in toluene were freshly prepared and used for L_van_Al(Et)K(Cp) (0.003 M) and 1,4-BDM (0.12 M). This sealed Schlenk was heated to 100 °C for 33 h. The polymerization was quenched by exposing the reaction mixture to air. PCHE-101 was purified by precipitation from DCM/MeOH (3x) and dried under vacuum at 40 °C. 1.5 g of the polymer was isolated (yield = 90%).

# NMR spectra of high molecular weight polyesters

Figure S 3. ^1^H NMR spectrum (CDCl_3_, 400 MHz, 298 K) of PCPE-75.

Figure S 4. ^1^H COSY NMR spectrum (CDCl_3_, 400 MHz, 298 K) of PCPE-75.

Figure S 5. ^1^H NMR spectrum (CDCl_3_, 400 MHz, 298 K) of PBE-77.

Figure S 6. ^1^H COSY NMR spectrum (CDCl_3_, 400 MHz, 298 K) of PBE-77.

Figure S 7. ^1^H NMR spectrum (CDCl_3_, 400 MHz, 298 K) of PeCHE-92.

Figure S 8. ^1^H COSY NMR spectrum (CDCl_3_, 400 MHz, 298 K) of PeCHE-92.

Figure S 9. ^1^H NMR spectrum (CDCl_3_, 400 MHz, 298 K) of PvCHE-98

Figure S 10. ^1^H COSY NMR spectrum (CDCl_3_, 400 MHz, 298 K) of PvCHE-98

Figure S 11. ^1^H NMR spectrum (CDCl_3,_ 400 MHz, 298 K) of PCHE-101.

Figure S 12. ^1^H COSY NMR spectrum (CDCl_3_, 400 MHz, 298 K) of PCHE-101.

# GPC traces of high molecular weight polyesters

Figure S 13. GPC trace (THF, 303 K) of PCPE-75.

Figure S 14. GPC trace (THF, 303 K) of PBE-77.

Figure S 15. GPC trace (THF, 303 K) of PeCHE-92.

Figure S 16. GPC trace (THF, 303 K) of PvCHE-98 before (black line) and after (red dots) processing.

Figure S 17. GPC trace (THF, 303 K) of PCHE-101.

At high polyester M_n_, there is a greater deviation between theoretical and experimental *M*_n_ values (see Table 1). This arises, in part, due to minor quantities of additional initiators (diol and diacid) in the monomers. These are demanding to remove, even with purification protocols, particularly since any water contamination can result in their formation from the pure monomers. These residual monomer impurities result in molecular weight values which deviate more substantially from theoretical values as the polymer molecular weight increases.

E.g. Table 1, entry 1 (cat:BDM:PA:CPO = 1:4:400:2000), 1.7 additional initiators can be determined:

$$\frac{\boldsymbol{400\cdot(148.1 kg/mol+84.1 kg/mol)}}{\boldsymbol{16200 kg/mol}}\boldsymbol{-4= 1.7}$$

At double the monomer loading (cat:BDM:PA:CPO = 1:4:800:4000), we would expect 3.4 additional initiators. The corrected *M*_n,theo_ = 25.1 kg mol^-1^ (which is very close to the value observed experimentally of 27.2 kg mol^-1^).

$$\frac{\boldsymbol{800\cdot(148.1 kg/mol+84.1 kg/mol)}}{\boldsymbol{7.4}}\boldsymbol{= 25100 kg/mol}$$

# Differential Scanning Calorimetry (DSC)

Figure S 18. Differential Scanning Calorimetry (DSC) data for PCPE-27.

Figure S 19. Differential Scanning Calorimetry (DSC) data for PCPE-40.

Figure S 20. Differential Scanning Calorimetry (DSC) data for PCPE-61.

Figure S 21. Differential Scanning Calorimetry (DSC) data for PCPE-75.

Figure S 22. Differential Scanning Calorimetry (DSC) data for PBE-77.

Figure S 23. Differential Scanning Calorimetry (DSC) data for PeCHE-92.

Figure S 24. Differential Scanning Calorimetry (DSC) data for PvCHE-98, stabilized with 0.1 wt % of a radical inhibitor, pentaerythritol tetrakis (3,5-di-tert-butyl-4-hydroxyhydrocinnamate) (PEHC).

Figure S 25. Differential Scanning Calorimetry (DSC) data for PCHE-101.

# Thermal Gravimetric Analysis (TGA)

Figure S 26. Thermogravimetric Analysis (TGA) data for PCPE-27.

Figure S 27. Thermogravimetric Analysis (TGA) data for PCPE-40.

Figure S 28. Thermogravimetric Analysis (TGA) data for PCPE-61.

Figure S 29. Thermogravimetric Analysis (TGA) data for PCPE-75.

Figure S 30. Thermogravimetric Analysis (TGA) data for PBE-77.

Figure S 31. Thermogravimetric Analysis (TGA) data for PeCHE-92.

Figure S 32. Thermogravimetric Analysis (TGA) data for PvCHE-98, stabilized with 0.1 wt % of a radical inhibitor, pentaerythritol tetrakis (3,5-di-tert-butyl-4-hydroxyhydrocinnamate) (PEHC).

Figure S 33. Thermogravimetric Analysis (TGA) data for PCHE-101.

# Rheology

## Temperature ramp experiments

Figure S 34. Oscillatory temperature ramp of PCPE-75 (1% strain, 1.0 Hz, 2 °C·min^-1^) between 100-160 °C. Top: dynamic moduli, *G*’ and *G*‘‘; bottom: tan(*δ*).

Figure S 35. Oscillatory temperature ramp of PBE-77 (1% strain, 1.0 Hz, 2 °C·min^-1^) between 50-140 °C. Top: dynamic moduli, *G*’ and *G*‘‘; bottom: tan(*δ*).

Figure S 36. Oscillatory temperature ramp of PeCHE-92 (1% strain, 1.0 Hz, 2 °C·min^-1^) between 110-210 °C. Top: dynamic moduli, *G*’ and *G*‘‘; bottom: tan(*δ*).

Figure S 37. Oscillatory temperature ramp of PvCHE-98, stabilized with 0.1 wt % of a radical inhibitor, pentaerythritol tetrakis (3,5-di-tert-butyl-4-hydroxyhydrocinnamate) (PEHC). (1% strain, 1.0 Hz, 2 °C·min^-1^) between 120-210 °C. Top: dynamic moduli, *G*’ and *G*‘‘; bottom: tan(*δ*).

Figure S 38. Oscillatory temperature ramp of PCHE-101 (1% strain, 1.0 Hz, 2 °C·min^-1^) between 120-210 °C. Top: dynamic moduli, *G*’ and *G*‘‘; bottom: tan(*δ*).

## Temperature Time Superposition (TTS) experiments

### Temperature Time Superposition (TTS) for PCPE-75

Figure S 39. Mastercurves of PCPE-75 constructed by time-temperature superposition (TTS) referenced to 170 °C (1% strain, 0.01-100 Hz). Top: dynamic moduli, *G*’ and *G*’’; bottom: tan(*δ*).

Figure S 40. Mastercurves of PCPE-75 constructed by time-temperature superposition (TTS) referenced to 170 °C (1% strain, 0.01-100 Hz): complex viscosity, *η**.

Figure S 41. Oscillatory frequency sweeps (1% strain) of PCPE-75 employed in the creation of mastercurves.

Figure S 42. Temperature dependence of the shift factors, αT, employed for the construction of the mastercurve of PCPE-75 (black diamonds) referenced at 170 °C and fitted curve (red line) to the WLF equation.

### Temperature Time Superposition (TTS) for PBE-77

Figure S 43. Mastercurves of PBE-77 constructed by time-temperature superposition (TTS) referenced to 140 °C (1% strain, 0.01-100 Hz). Top: dynamic moduli, *G*’ and *G*’’; bottom: tan(*δ*).

Figure S 44. Mastercurves of PBE-77 constructed by time-temperature superposition (TTS) referenced to 170 °C (1% strain, 0.01-100 Hz): complex viscosity, *η**.

Figure S 45. Oscillatory frequency sweeps (1% strain) of PBE-77 employed in the creation of mastercurves.

Figure S 46. Temperature dependence of the shift factors, αT, employed for the construction of the mastercurve of PBE-77 (black diamonds) referenced at 140 °C and fitted curve (red line) to the WLF equation.

### Temperature Time Superposition (TTS) for PeCHE-92

Figure S 47. Mastercurves of PeCHE-92 constructed by time-temperature superposition (TTS) referenced to 170 °C (1% strain, 0.01-100 Hz). Top: dynamic moduli, *G*’ and *G*’’; bottom: tan(*δ*).

Figure S 48. Mastercurves of PeCHE-92 constructed by time-temperature superposition (TTS) referenced to 170 °C (1% strain, 0.01-100 Hz): complex viscosity, *η**.

Figure S 49. Oscillatory frequency sweeps (1% strain) of PeCHE-92 employed in the creation of mastercurves.

Figure S 50. Temperature dependence of the shift factors, αT, employed for the construction of the mastercurve of PeCHE-92 (black diamonds) referenced at 170 °C and fitted curve (red line) to the WLF equation.

### Temperature Time Superposition (TTS) for PvCHE-98

Figure S 51. Mastercurves of PvCHE-98 (stabilized with 0.1 wt % of a radical inhibitor, pentaerythritol tetrakis (3,5-di-tert-butyl-4-hydroxyhydrocinnamate) (PEHC)) constructed by time-temperature superposition (TTS) referenced to 170 °C (1% strain, 0.01-100 Hz). Top: dynamic moduli, *G*’ and *G*’’; bottom: tan(*δ*).

Figure S 52. Mastercurves of PvCHE-98 constructed by time-temperature superposition (TTS) referenced to 170 °C (1% strain, 0.01-100 Hz): complex viscosity, *η**.

Figure S 53. Oscillatory frequency sweeps (1% strain) of PvCHE-98 employed in the creation of mastercurves.

Figure S 54. Temperature dependence of the shift factors, αT, employed for the construction of the mastercurve of PvCHE-98 (black diamonds) referenced at 170 °C and fitted curve (red line) to the WLF equation.

### Temperature Time Superposition (TTS) for PCHE-101

Figure S 55. Mastercurves of PCHE-101 constructed by time-temperature superposition (TTS) referenced to 170 °C (1% strain, 0.01-100 Hz). Top: dynamic moduli, *G*’ and *G*’’; bottom: tan(*δ*).

Figure S 56. Mastercurves of PCHE-101 constructed by time-temperature superposition (TTS) referenced to 170 °C (1% strain, 0.01-100 Hz): complex viscosity, *η**.

Figure S 57. Oscillatory frequency sweeps (1% strain) of PCHE-101 employed in the creation of mastercurves.

Figure S 58. Temperature dependence of the shift factors, αT, employed for the construction of the mastercurve of PCHE-101 (black diamonds) referenced at 170 °C and fitted curve (red line) to the WLF equation. Dynamic Mechanical Analyzer (DMA)

## Dynamic Mechanical Thermal Analysis (DMTA)

Figure S 59. Dynamic mechanical temperature analysis (DMTA) temperature sweeps for PCPE-75. Lower figure compares the different Tg values determined for this sample by DSC (blue), DMTA (red) and rheology (black).

Figure S 60. Dynamic mechanical temperature analysis (DMTA) temperature sweeps for PBE-77.

Figure S 61. Dynamic mechanical temperature analysis (DMTA) temperature sweeps for PeCHE-92.

Figure S 62. Dynamic mechanical temperature analysis (DMTA) temperature sweeps for PvCHE-98, stabilized with 0.1 wt % of a radical inhibitor, pentaerythritol tetrakis (3,5-di-tert-butyl-4-hydroxyhydrocinnamate) (PEHC).

Figure S 63. Dynamic mechanical temperature analysis (DMTA) temperature sweeps for PCHE-101.

## 3-Point bend experiments

Figure S 64. 3-Point bend dynamic mechanical analysis (DMA) data for PCPE-27.

Figure S 65. 3-Point bend dynamic mechanical analysis (DMA) data for PCPE-40.

Figure S 66. 3-Point bend dynamic mechanical analysis (DMA) data for PCPE-75.

Figure S 67. 3-Point bend dynamic mechanical analysis (DMA) data for PBE-77.

Figure S 68. 3-Point bend dynamic mechanical analysis (DMA) data for PeCHE-92.

Figure S 69. 3-Point bend dynamic mechanical analysis (DMA) data for PvCHE-98, stabilized with 0.1 wt % of a radical inhibitor, pentaerythritol tetrakis (3,5-di-tert-butyl-4-hydroxyhydrocinnamate) (PEHC).

Figure S 70. 3-Point bend dynamic mechanical analysis (DMA) data for PCHE-101.

# Tensile Test

Figure S 71. Tensile strain-stress plots for PCPE-75.

Figure S 72. Tensile strain-stress plots for PBE-77.

Figure S 73. Tensile strain-stress plots for PvCHE-98, stabilized with 0.1 wt % of a radical inhibitor, pentaerythritol tetrakis (3,5-di-tert-butyl-4-hydroxyhydrocinnamate) (PEHC).

Figure S 74. Tensile strain-stress plots for PCHE-101.

# Hydrogenation of PvCHE-91

The unsaturated polyester PvCHE-92 (800 mg) was dissolved in THF (20 mL) and mixed with 10 wt% Pd/C. The suspension was stirred at 40 °C and 40 bar H_2_ in a Parr reactor for 24 h. The polymer was purified by filtration and sequential precipitation from DCM/methanol.

Figure S 75. GPC trace (THF, 303 K) for BDM-initiated PvCHE-75, before (dark grey) and after (red) hydrogenation (in THF, 40 bar H_2_, 40 °C).

Figure S 76. ^1^H NMR spectrum (CDCl_3_, 298 K) for CHD-initiated PvCHE-91, before (bottom) and after (top) hydrogenation (in THF, 40 bar H_2_, 40 °C).

Figure S 77. GPC trace (THF, 303 K) for CHD-initiated PvCHE-92, before (dark grey) and after (orange) hydrogenation (in THF, 40 bar H_2_, 40 °C).

# Recycling Study

## Procedure

After mechanical testing, PBE-77 was mechanically recycled. The specimens were compression molded at 70 °C to a smooth film. Sequential testing was conducted according to ISO 527, using minimum 4 dumbbell-shaped specimens, according to ISO 527-2 type 5B.


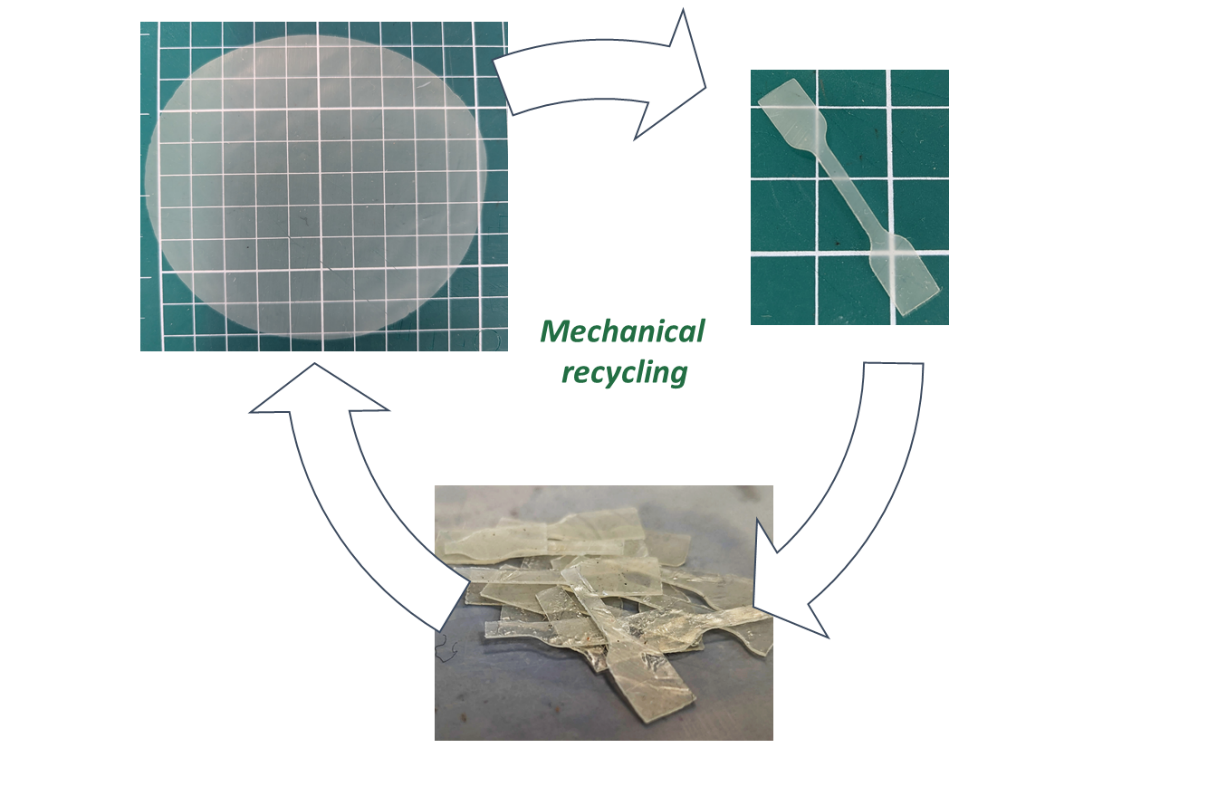


Figure S 78. Pictures of mechanical recycling.

## Tensile Test of Recycled Polymer

Figure S 79. Tensile strain-stress plots for the recycling of PBE-77. A) first recycling, B) second recycling, C) third recycling, D) fourth recycling.

## GPC of Recycled Polymer

Figure S 80. GPC trace (THF, 303 K) for PBE-77, before (dark grey) and after (red) recycling (compression moulding at 70 °C, 1 h).

## NMR of Recycled Polymer

Figure S 81. ^1^H NMR spectrum (CDCl_3_, 298 K) for PBE-77, before (bottom) and after (top) recycling (compression moulding at 70 °C, 1 h).

# Commercial comparison

## Ashby Plot

Data used for the Ashby plot was aquired from online databases.^[4]^

Table S 1. Tensile and thermal properties of polystyrene.^[4]^

| **Polymer grade** | **Tensile Strain (Yield) / MPa** | **Tensile Strain (Break) / MPa** | **Elongation at Break / %** | ***T*_g_ / °C** |
| --- | --- | --- | --- | --- |
| molded unreinforced | 32.3 | 41 | 13.7 | 90.5 |
| Extrusion grade | 34.9 | 22.9 | 21 | - |
| impact modified | 25.8 | 26.9 | 45.2 | - |
| heat resistant | 45.9 | 47.1 | 9.16 | 98 |
| transparent grade | 43.6 | 46.1 | 3.14 | - |
| Aclo Accucomp Polystyrene GS0500L Low Impact | - | 45.0 | 5.0 | 93.3 |
| Aclo Accucomp Polystyrene HS0600L High Impact | - | 24.8 | 25 | 82.2 |
| Mocom (ALBIS) ALCOM® MED PS 1000 04001 PS | - | 45.0 | 1.5 | - |
| American Polymers API 375 Polystyrene |  | 44.5 | 1.5 | 102 |
| American Polymers 370 Polystyrene, High Heat | - | 50.7 | 3.0 | 95.0 |

Table S 2. Tensile and thermal properties of polylactide.^[4]^

| **Polymer grade** | **Tensile Strain / MPa** | **Elongation at Break / %** | **Elongation at Yield / %** | ***T*_g_ / °C** | ***T*_m_ / °C** |
| --- | --- | --- | --- | --- | --- |
| Cereplast Compostables® 7003 | 55.2 | 7 | 3.45 | - | - |
| Ingeo™ 2500HP | 64.1 | 3.6 | - | - | 173 |
| Ingeo™ 4060D | - | - | - | 55 | - |
| LACTEL® L-PLA - PLLA | 68 | 7.5 | 3.45 | 62 | 175 |
| Luminy® L175 - PLLA | 50 | 5 | 3.5 | 60 | 175 |
| Purac PLA Blend A - genral purpose | 45 | 5 | 3 | - | - |
| Pure PLA 120 | 63 | 2.75 | 3.25 | 63 | 175 |
| REVODE 201 | 45 | 3 | - | 57 | 143 |

Figure S 82. Ashby plot from data for different grades of PS and PLA from online databases.^[4]^

## Rheology

Commercial polystyrene samples (*M*_w_ = 38 kg·mol^-1^ and *M*_w_ = 280 kg mol^-1^) were investigated by oscillatory rheology. Temperature ramp experiments (1% strain, 1.0 Hz, 2 °C·min^-1^) were conducted and the complex viscosity of bith samples was compared to PCPE-75.

Figure S 83. Oscillatory temperature ramp of PS-38 (1% strain, 1.0 Hz, 2 °C·min^-1^) between 120-210 °C. Top: dynamic moduli, *G*’ and *G*‘‘; bottom: tan(*δ*).

Figure S 84. Oscillatory temperature ramp of PS-280 (1% strain, 1.0 Hz, 2 °C·min^-1^) between 120-210 °C. Top: dynamic moduli, *G*’ and *G*‘‘; bottom: tan(*δ*).

Figure S 85. Complex viscosity from oscillatory temperature ramp of PS-38, PS280 and PCPE-75 (1% strain, 1.0 Hz, 2 °C·min^-1^) between 65-220 °C.

# Additional Tables

Table S 3. Synthesis of polyesters.^[a]^

| **Epoxide** | **Ester selectivity^[b]^/%** | **TOF^[b]^/ h^-1^** | ***M*_n,GPC_^[c]^ / kg·mol^-1^ [*Đ*]^[d]^** | ***M*_n,th_^[e]^ / kg·mol^-1^** |
| --- | --- | --- | --- | --- |
| CHO^[3]^ | 95 | 1032 | 20.1 [1.10] | 24.8 |
| vCHO^[3]^ | >99 | 528 | 24.8 [1.05] | 27.4 |
| CPO | >99 | 514 | 16.2 [1.04] | 23.3 |
| BO | >99 | 224 | 20.5 [1.05] | 22.2 |

[a] Conditions: [1] : [BDM] : [PA] : [epoxide] = 1 : 4 : *x* : *y* where *x* is given and *x* : *y* = 1 : 5, *T* = 100 ºC. [b] Determined with ^1^H NMR spectroscopy. [c] Determined by GPC in THF, at 30 ºC, using narrow dispersity polystyrene standards. [d] Dispersity = *M*_w_/*M*_n_, determined by GPC in THF, at 30 °C. [e] Theoretical molar mass, determined by (TON · *M*_n,repeat unit_/4) + *M*_n,BDM._

Table S 4. Thermal properties of poly(PA-*alt*-CPO)

| **Polymer** | ***T*_g,DSC_^[a]^ / °C** | ***T*_d, 5%_^[b]^ / °C** |
| --- | --- | --- |
| PCPE-27 | 96 | 300 |
| PCPE-40 | 92 | 298 |
| PCPE-61 | 97 | 293 |
| PCPE-75 | 94 | 293 |

[a] ] Measured by DSC from the second scan at a heating rate of 10 °C·min^-1^. [b] Onset of thermal decomposition, as measured by TGA.

Table S 5**.** DMTA data for high molecular weight polyesters.^[a]^

| **polymer** | ***T*_plat_^[b]^ / ^o^C** | ***E*^0^_N_^[c]^ / MPa** | ***G*^0^_N,DMTA_^[d]^ / MPa** | ***M*_e, DMTA_^[e]^ / kg·mol^-1^** |
| --- | --- | --- | --- | --- |
| PCPE-75 | 112.7 | 0.4920 | 0.181 | 11-16 |
| PBE-77 | 83.8 | 0.4310 | 0.159 | 12-17 |
| PCHE-101 | 148.4 | 0.6129 | 0.225 | 10-14 |
| PvCHE-98**^[e]^** | 148.1 | 0.6707 | 0.247 | 9-13 |
| PeCHE-92 | 151.4 | 0.1835 | 0.068 | 33-46 |

Table S 6. Mechanical properties of polyesters: tensile data and 3-point bend DMA.

| **polymer** | ***σ*_f_^[a]^ / MPa** | ***ɛ*_f_ ^[b]^ / %** | ***E*_f_ ^[c]^ / GPa** | ***σ* ^[d]^ / MPa** | ***ɛ*^[e]^ / %** | ***E*_y_^[f]^ / GPa** | ***u*_t_^[g]^ / MJ·m^−3^** |
| --- | --- | --- | --- | --- | --- | --- | --- |
| PCPE-27 | 16.5 ± 4.9 | 0.8 ± 0.2 | 2.3 ± 0.3 | - | - | - | - |
| PCPE-40 | 52.0 ± 3.5 | 2.8 ± 0.6 | 2.6 ± 0.3 | 42.4 ± 9.9 | 4.5 ± 0.9 | 1.08 ± 0.42 | 1.1 ± 0.3 |
| PCPE-61 | 77.0 ± 3.9 | 5.2 ± 0.5 | 2.3 ± 0.5 | 43.3 ± 5.7 | 5.8 ± 0.9 | 1.05 ± 0.10 | 1.5 ± 0.4 |
| PCPE-75 | 82.0 ± 3.2 | 6.0 ± 0.8 | 3.0 ± 0.3 | 47.2 ± 1.4 | 7.2 ± 1.6 | 0.94 ± 0.03 | 2.1 ± 0.6 |

[a] Ultimate flexural strength. [b] Flexural strain at break. [c] Flexural modulus, determined from the strain/stress gradient. [d] Ultimate tensile strength. [e] Tensile strain at break. [f] Young‘s modulus, determined from the strain/stress gradient. [g] Tensile toughness.

# Additional Figures

Figure S 86. GPC traces (THF, 303 K) for poly(PA-*alt*-CPO) during the reaction.

Figure S 87. *M*_n_ vs. conversion plot for poly(PA-*alt*-CPO).

Figure S 88. ^1^H NMR spectrum (CDCl_3_, 298 K) for the ring-opening copolymerization of CHO and PA with L_van_Al(Et)K(Cp) and BDM (cat:BDM:PA:CHO = 1:4:3200:16000), performed in neat CHO.

Figure S 89. GPC trace (THF, 303 K) for poly(PA-*alt*-CHO), polymerization performed in neat CHO.


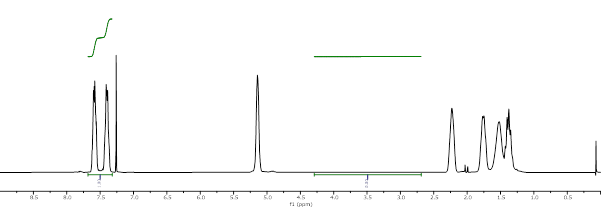


Figure S 90. ^1^H NMR spectrum (CDCl_3,_ 400 MHz, 298 K) of PCHE-101: integration of the ether region.

# References

[1] A. Thevenon, J. A. Garden, A. J. White, C. K. Williams, *Inorg. Chem.* **2015**, *54*, 11906-11915.

[2] W. T. Diment, T. Stößer, R. W. F. Kerr, A. Phanopoulos, C. B. Durr, C. K. Williams, *Catal. Sci. Technol.* **2021**, *11*, 1737-1745.

[3] W. T. Diment, C. K. Williams, *Chem. Sci.* **2022**, *13*, 8543-8549.

[4] *MatWeb - Material Property Data*, can be found under: <https://www.matweb.com/> (accessed: 09/01/2025).
